# Supplementary material for: Anthocyanin Biosynthesis Associated with Natural Variation in Autumn Leaf Coloration in Quercus aliena Accessions
Source: Int J Mol Sci. 2022 Oct 12;23(20):12179. doi: 10.3390/ijms232012179 (PMC9603646; doi:10.3390/ijms232012179)
Supplement: Supplementary file 1 [file ijms-23-12179-s001.zip › ijms-1939711-supplementary/Supplementary materials.pdf]

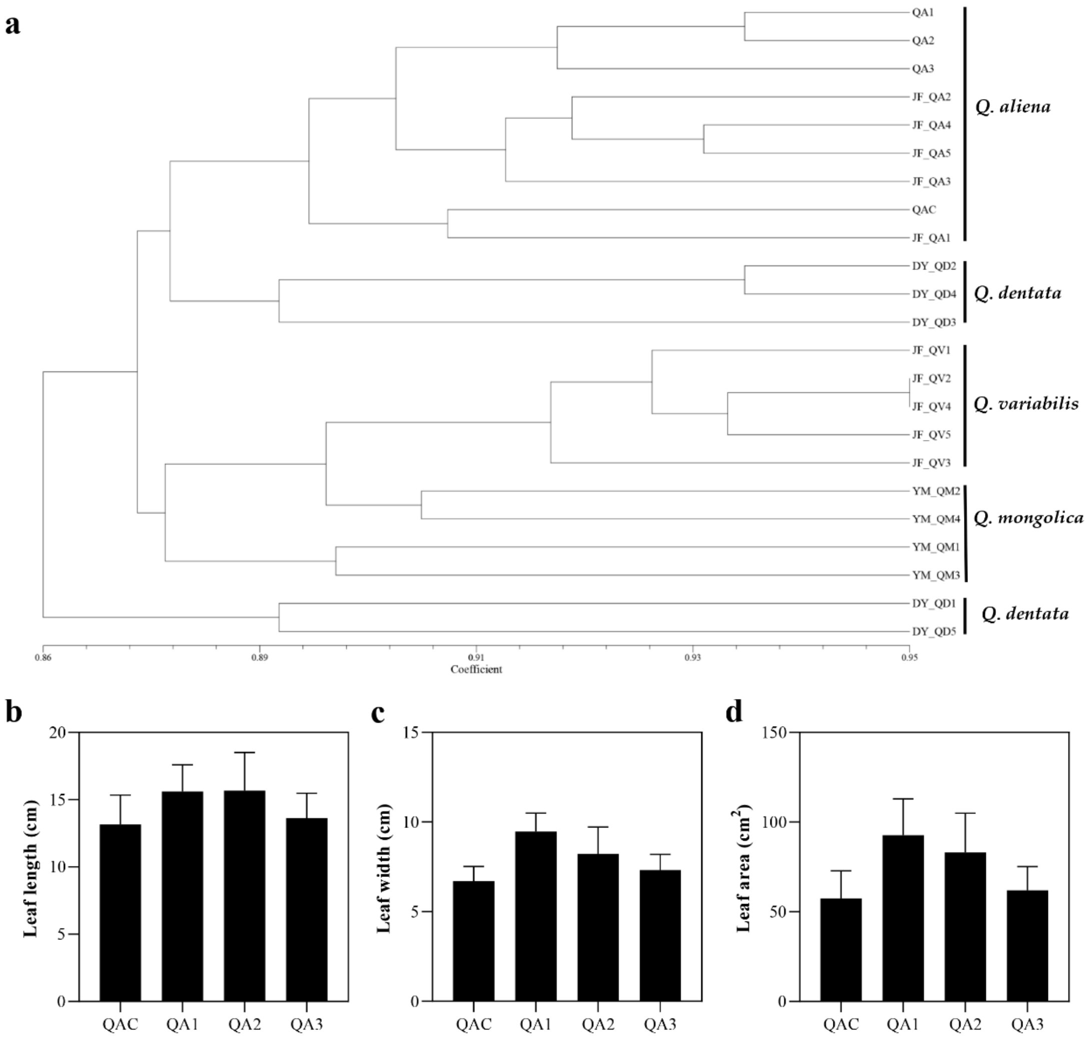

Figure S1. Analysis of relationship and leaves phenotypic in *Q. aliena* accessions. (**a**) JF_QA1, JF_QA2, JF_QA3, JF_QA4, and JF_QA5 belong to *Q. aliena*, and are planted in the Jiufeng National Forest Park, Beijing, China. DY_QD1, DY_QD2, DY_QD3, DY_QD4, and DY_QD5 belong to *Q. dentata*, and are planted in the Dayangshan National Forest Park, Beijing, China. YM_QM1, YM_QM2, YM_QM3, and YM_QM4 belong to *Q. mongolica*, and are planted in the Yunmengshan National Forest Park, Beijing, China. JF_QV1, JF_QV2, JF_QV3, JF_QV4, and JF_QV5 belong to *Q. variabilis*, and are planted in the Jiufeng National Forest Park, Beijing, China. (**b**)-(**d**) leaves phenotypic of the four trees at S3 stage, bars represent the standard deviation (SD, n = 15).

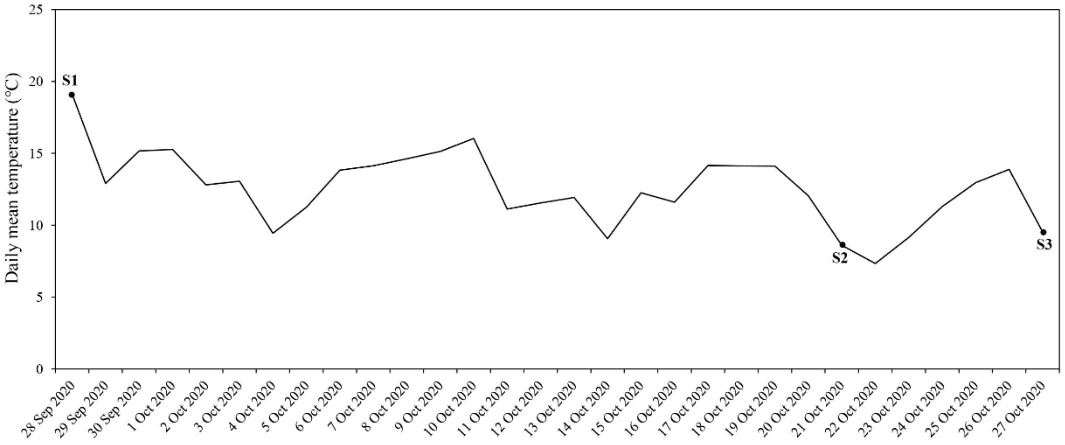

Figure S2. Daily mean temperatures in Beijing during the discoloration period (Sep-Oct 2020).

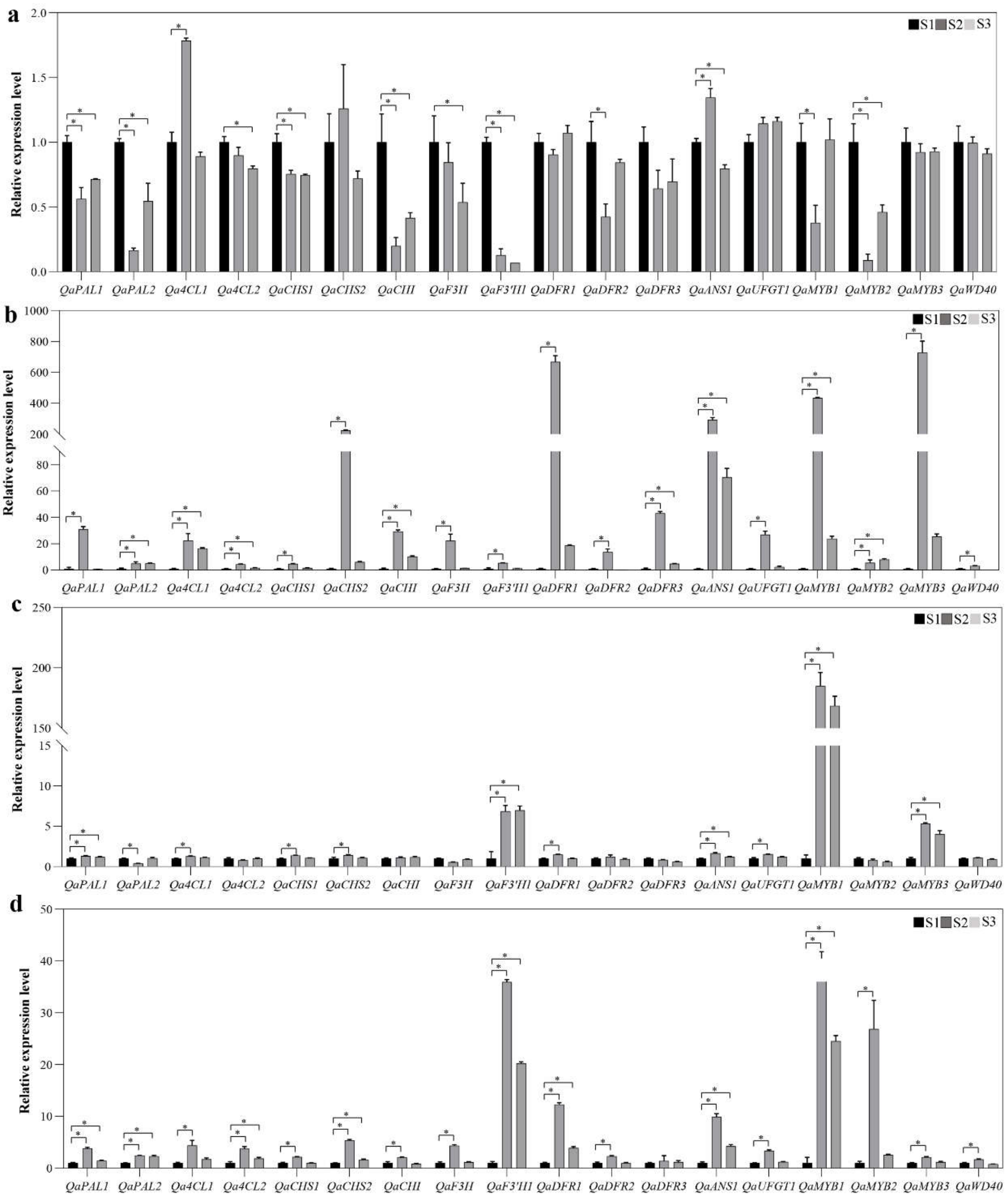

Figure S3. Gene expression during leaf senescence based on semi-quantitative PCR: (a) QAC, (b) QA1, (c) QA2, and (d) QA3. Bars represent standard deviation (SD). * $P < 0.05$.

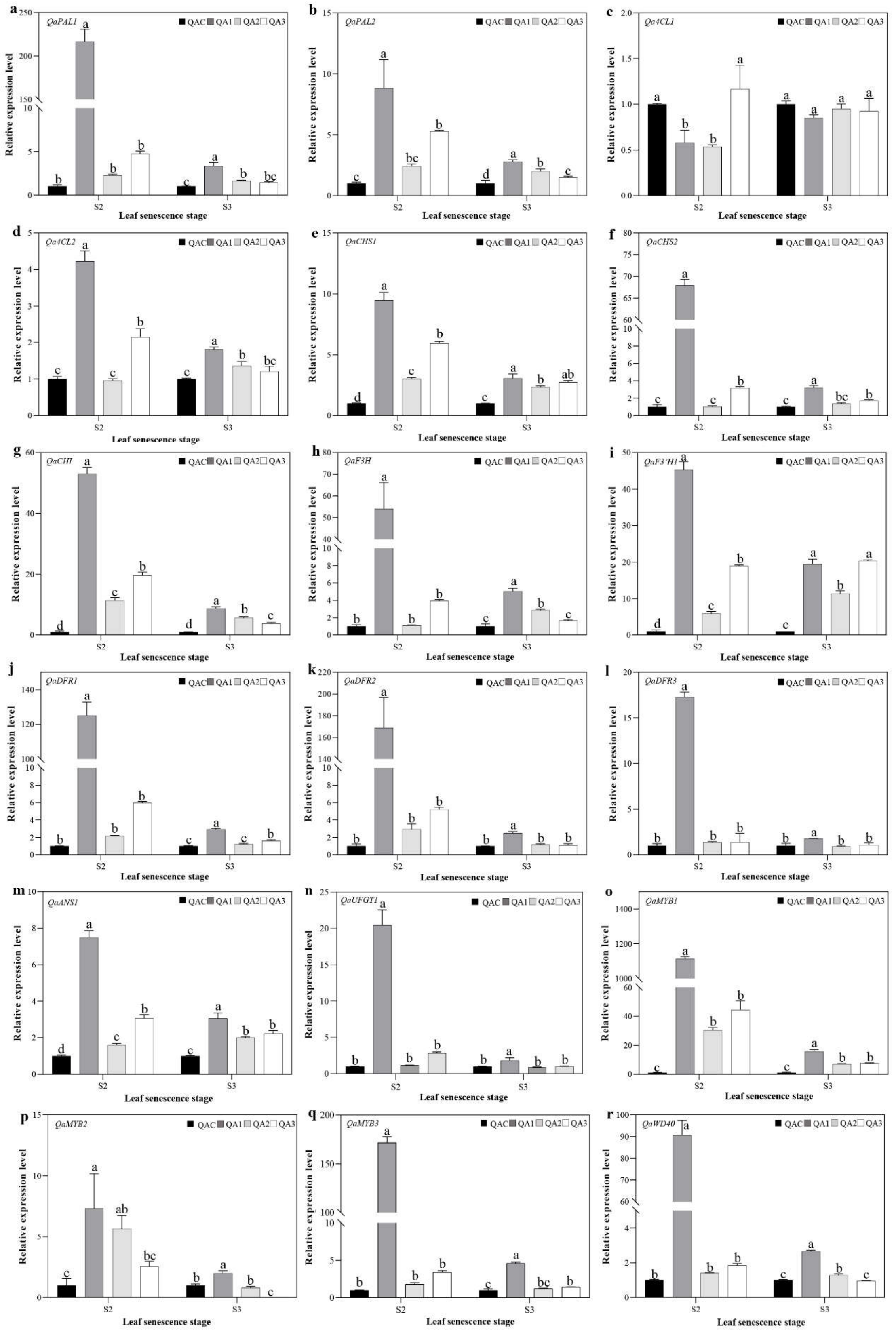

Figure S4. Differential gene expression during leaf senescence in *Q. aliena* accessions based on semi-quantitative PCR. Bars represent standard deviation (SD). * $P < 0.05$, and lowercase letters represent significant differences at $P < 0.05$.

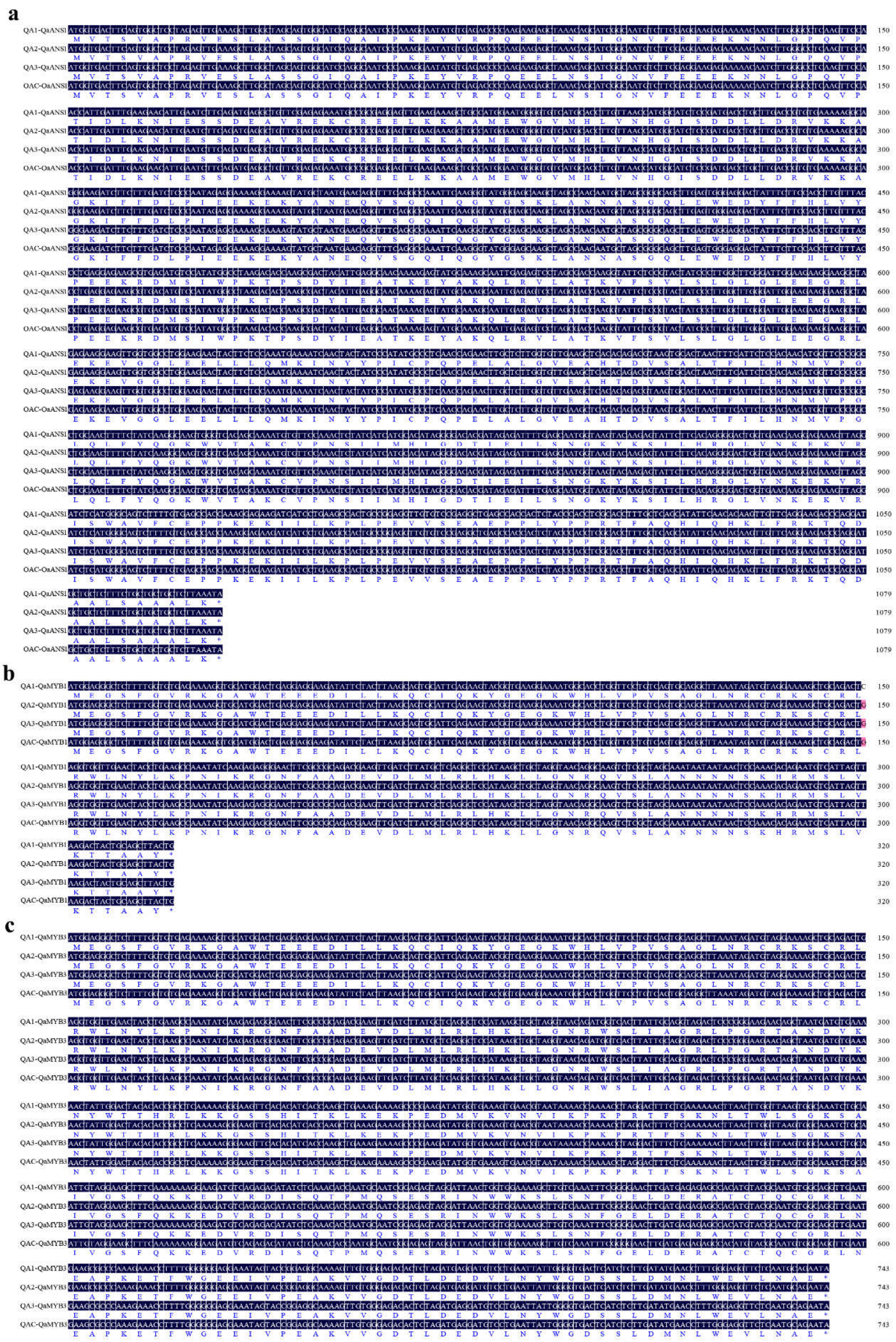

Figure S5 Homology analysis of *QaANS1* (a), *QaMYB1* (b), and *QaMYB3* (c) in the three variants and QAC.

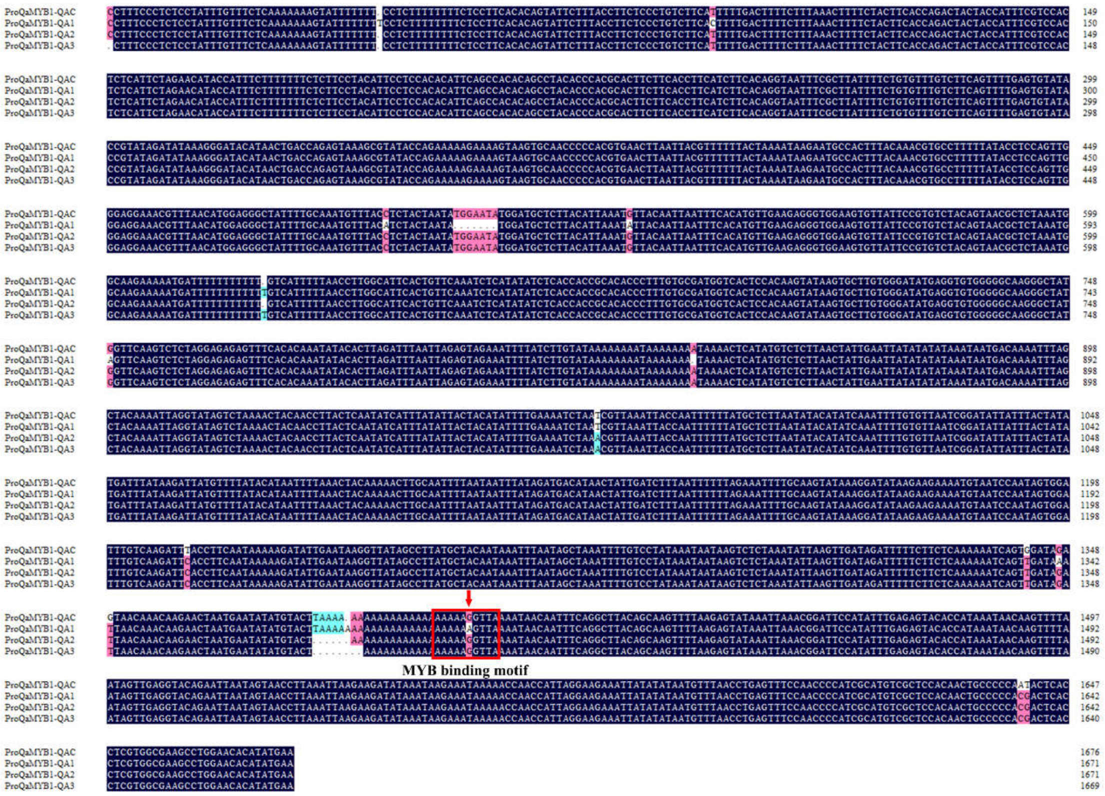

Figure S6 Analysis of *QaMYB1* promoter regions in *Q. aliena* accessions.

Table S1. Anthocyanin synthesis-related genes in Quercus aliena and other species

**Gene**	**Homologue in Q. robur L.**	**Homologues in other species (gene ID)**
QaPAL1	Qrob_P0101950.2	*AtPAL1* (AT2G37040), *AtPAL2* (AT3G53260)
QaPAL2	Qrob_P0328050.2	*AtPAL3* (AT5G04230), *AtPAL4* (AT3G10340)
Qa4CL1	Qrob_P0278330.2	
Qa4CL2	Qrob_P0058290.2	*At4CL1* (AT1G51680), *At4CL2* (AT3G21240)<br>*At4CL3* (AT1G65060)
Qa4CL3	Qrob_P0766030.2	
QaCHS1	Qrob_P0522690.2	
QaCHS2	Qrob_P0004890.2	*AtCHS* (AT5G13930)
QaCHS3	Qrob_P0063990.2	
QaCHS4	Qrob_P0276680.2	
QaCHI	Qrob_P0211560.2	*AtCHI* (AT3G55120)
QaF3H	Qrob_P0200790.2	*AtF3H* (AT3G51240)
QaF3′H1	Qrob_P0098800.2	*VvF3′H* (NC_012023.3)
QaF3′H2	Qrob_P0276000.2	
QaDFR1	Qrob_P0599010.2	
QaDFR2	Qrob_P0497760.2	*AtDFR* (AT5G42800)
QaDFR3	Qrob_P0119180.2	
QaANS1	Qrob_P0502480.2	*AtANS* (AT4G22880)
QaANS2	Qrob_P0514540.2	
QaUFGT1	Qrob_P0392850.2	
QaUFGT2	Qrob_P0624920.2	*VvUFGT* (NC_012022.3)
QaUFGT3	Qrob_P0624910.2	
QaMYB1	Qrob_P0355830.2	
QaMYB2	Qrob_P0437590.2	*VvMYBA1* (BAD18977.1), *VvMYBA2* (BAD18978.1)<br>*MdMYB10* (DQ267896), *FaTTG1* (JQ989287)<br>*DcTT8* (PKU66073.1)
QaMYB3	Qrob_P0398080.2	
QaWD40	Qrob_P0231390.2	

Table S2. Primers used in the study

Gene	Forward primer	Reverse primer
Primers used for PCR amplification		
QaPAL1	ATGGAATATTGCAATGGAA	TTAATTTATAGGAAGAGGAGCAC
QaPAL2	ATGGGAACCAATATTAATAAAAG	TCACCTAATCAACTCGGTC
Qa4CL1	ATGGAGTCCCAAAAAGACCT	TTAATTTGCTAGACCTGCTGC
Qa4CL2	ATGGCTCTCCAAACAAAGGA	TCAAAGGTGAAAGGGACCC
Qa4CL3	ATGGGAACTTATTTGCATAATTT	TTATTTTTCAACATTGGATTCG
QaCHS1	ATGGCTTCGTCTTTGGATG	TCAGTGACTGGTTTCCACG
QaCHS2	ATGGTGACTGTTGATGAAGTACG	TTAAGCAGAGACGCTGTGGA
QaCHS3	ATGTCAAGAACTAATGGCAATG	TTACAGGCTACGGACGAGTAT
QaCHS4	ATGGCGTTAGTTGGAAATCTC	TCACCCATGCTTTATAGGAGAA
QaCHI1	ATGGTCCTAGTAGCGTCTCTTC	TCAGTGACCTTCACATTCACA
QaF3H	ATGGCTCCTACTACTCTCACAGC	CTAAGCAAAGATATCCTCAACGG
QaF3′H1	ATGGAGTCTTCCTCTTCTTGG	CTAATAAAGATGAAGCGGGAG
QaF3′H2	ATGGACTTTTGGATCTTTGTAAT	TTAGAAAGTAATAGGAATAGCTT
QaDFR1	ATGGGGTCAGAGGGTGAG	TCAATCTTTTTTCGTGCCAG
QaDFR2	ATGGAAGGAGATAAGGGTACAGT	CTAGAGAAACCCCTTTTCTTTG
QaDFR3	ATGGCCACCCAAAACCTT	TTATATCTGCAGCAGGCCCT
QaANS1	ATGGTGACTTCAGTGGCTC	TTATTTAAGAGCAGCAGCAGA
QaANS2	ATGGAACCAGAAATAACGACG	TTAAGTGCCTTTCTGGATCCTC
QaUFGT1	ATGTCCTCTTCCTCGACCAAT	TCATTCCTTTGCTGCATTTG
QaUFGT2	ATGTCGATGACCAAAAGCTC	TTAAGGCACAGATATTAGCTCCA
QaUFGT3	ATGTCAGTGACCAAAAGCTCTC	TTAAGGCACAGATGTTAGCTCG
QaMYB1	ATGGAGGGCTCTTTCGGT	TCAGTAAGCTGCAGTAGTCTTAA
QaMYB2	ATGGGAAGGGCTCCTTGT	TCAGATCAATAGTGATTCAGCAA
QaMYB3	ATGGAGGGCTCTTTTGGT	CTATTCTGCATTGAGAACCTCC
QaWD40	ATGGGTGCGAGTAGCGAT	TCATACCCTAAGTATCTGAAGCT
Primers used for semi-quantitative or quantitative real-time PCR		
YGQaPAL1	CTTGGCCCACAAATTGAAGT	TGAAGCAATGGCTAGACGTG
YGQaPAL2	GTGAAGCTCGGAGGTGAGAC	GAAGTTGCACCAAACCCAGT
YGQa4CL1	GCCAAACTTGGACAGGGTTA	CCAGTTTCTGGGTCAACGAT
YGQa4CL2	GGCATTGGACAAGGAGATGT	TGCGTGATAATGAGCTTTGC
YGQaCHS1	AGTTGGGAAAAGAAGCAGCA	CAGCAAAGCAACCTTGTTGA
YGQaCHS2	CCCAATTCCTGAGGTTGAGA	TTTGAAATGAGGCCAGGAAC
YGQaCHI	GAAAGAGTGCGGAGGAGTTG	TTTTCAACGGCTTTGGTTTC
YGQaF3H	GGGAGAAGACCCTTCAGTCC	AACACCCCAGTCCTCACAAG
YGQaF3′H1	CAGGGCGGAGATTGATAAAA	GTATTGCCTCTGCCAATGGT
YGQaDFR1	GAGGGTGAGACCGTTTGTGT	CTTCGTTCAGGTCAGCCTTC
YGQaDFR2	GGCCATAACACTCCAAAGGA	TTGAATGGCTCCATCAAACA
YGQaDFR3	CACATGTGGAGGATGTTTGC	GGGAAATCCCCAAAATCAGT
YGQaANS1	TGGGTCACAGCAAAATGTGT	TGGTGGCTCACAAAAGACTG
YGQaUFGT1	CAAACATGGTGGTGAACTGC	ACTCACCTCCTTCCCCAACT
YGQaMYB1	TTCCTGTCAGTGCAGGCTTA	GCTAGCGAGACTTGCCTGTT

YGQaMYB2	GGCTGCTTAGGTGTGGAAAG	TTATCAGTTCGACCGGGAAG
YGQaMYB3	TCCGGGAAGAACAGCTAATG	GCAGGTTTCCCACTTAACCA
YGQaWD40	GAGACACCAAGCGTCTGTCA	CTGAGACGACGACCACTGAA
Primers used for analysis of simple sequence repeats		
ssrQrZAG96	CCCAGTCACATCCACTACTGTCC	GGTTGGGAAAAGGAGATCAGA
ssrQrZAG102	GCCTACACTCTTCAATCTACATGA	GACTTGTAACACCTTAAGCATTATCT
ssrQrZAG112	TTCTTGCTTTGGTGCGCG	GTGGTCAGAGACTCGGTAAGTATTC
ssrQrZAG7	CAACTTGGTGTTCGGATCAA	GTGCATTTCTTTTATAGCATTCAC
Qden03011	AACCCAACCTTCCCTTCATC	GCAGTGGTGCCTAATGTAGAC
Qden03021	ACAGCAAACCAGACTCCAC	CCCCAAAGTTTCGGCTAATAC
Qden03032	AGTTGTGGTCCTGCTCGC	GAAAAGTGCGATGACGGTTG
Qden05011	CCCACTCCCTGTCCATTGT	CACTGTGTGCTGCGACTTG
Qden05031	CCCCGATTCGCCATCATTGT	GTAACGCCGTTTTTCTCCACC
